# Draft Genome Sequences of Mexican Babesia bovis Virulent and Attenuated Strains

**DOI:** 10.1128/mra.01153-21

**Published:** 2022-03-09

**Authors:** Bernardo Sachman-Ruiz, Luis Lozano, José J. Lira, Grecia Martínez, Carmen Rojas, J. Antonio Álvarez, Julio V. Figueroa

**Affiliations:** a Centro Nacional de Investigación Disciplinaria en Salud Animal e Inocuidad, INIFAP, Jiutepec, Morelos, México; b Centro de Ciencias Genómicas, UNAM, Cuernavaca, Morelos, México; Vanderbilt University

## Abstract

Babesia bovis, a tick-borne intraerythrocytic protozoan parasite that belongs to the phylum Apicomplexa, is one of the etiological agents of bovine babesiosis, a highly prevalent disease in tropical and subtropical countries that causes significant morbidity and deaths in cattle. This report presents the draft genome sequences of attenuated and virulent B. bovis strains of Mexican origin.

## ANNOUNCEMENT

Cattle infected with virulent Babesia bovis strains clinically manifest hemolytic anemia and cerebral babesiosis (1, 2). Under *in vitro* culture conditions, attenuated strains of *Babesia* species have been attained (3–5). Culture-derived *Babesia* parasites are less virulent, are able to induce immunity in cattle challenged with virulent strains (6), and are not transmitted by ticks to susceptible cattle (7). To compare the genomes of virulent and attenuated B. bovis strains, the objective of this study was to assemble a draft genome for each parasite population. The attenuated strain was derived from *in vitro* culture using a microaerophilic stationary system (6) and has been maintained alternately in continuous culture and cryopreservation in liquid nitrogen at −196°C at Centro Nacional de Investigación Disciplinaria en Salud Animal e Inocuidad (CENID-SAI) (6–9).

Briefly, a cryostabilate of an attenuated B. bovis strain was thawed at 37°C and suspended in VyM solution (9). After centrifugation at 450 × *g* for 30 min at 25°C, 1.0 mL of a 10% bovine erythrocyte suspension in culture medium M-199 supplemented with 40% bovine serum was added to the B. bovis pellet. The mixture was transferred to 24-well tissue culture plates and incubated at 37°C in an atmosphere of 90% N_2_, 5% O_2_, and 5% CO_2_ at constant pressure. The culture medium was replaced every 24 h, and a subculture was established when the percentage of parasitized erythrocytes (PPE) reached 4% or higher, by adding a suspension of erythrocytes at 10% and transferring the suspension to a 50-mL culture flask. The virulent strain has been maintained through tick passages in susceptible animals and cryopreservation in liquid nitrogen at CENID-SAI (1, 6, 7). A 5-mL cryostabilate of a virulent B. bovis strain with 2% PPE was thawed at 37°C and reactivated in a splenectomized calf as before (6) for B. bovis-infected erythrocyte collection. Bovine erythrocytes infected with the two populations of B. bovis were subjected to extraction of genomic DNA by traditional organic phenol-chloroform extraction protocols (10, 11). Twenty to 30 μg of genomic DNA was used in the whole-genome sequencing project performed at the Sequencing Core Facility of the National Autonomous University of Mexico (IBT-UNAM). Two DNA libraries were constructed for each parasite population by using the Nextera XT DNA library preparation kit (Illumina, San Diego, CA). The genomes were assembled using SPAdes v3.13.1 (12) with the parameters --careful, –k 21,31,41,51,61,71, yielding 5,239,525 pairs of paired-end reads for the attenuated strain and 8,237,626 pairs of paired-end reads for the virulent strain. Quality control was performed with Trim Galore software v0.6.4 using default parameters. A total of 625 contigs with 480× coverage for the attenuated strain and a total of 2,274 contigs with 130× coverage for the virulent strain were used for mapping of the sequences against a reference genome sequence, i.e., B. bovis T2Bo strain (BioProject accession number PRJNA18731). The mapping was performed with nucleotide MUMmer system software v3.1 with default parameters. To eliminate bovine DNA contamination, sequence alignments were performed with the Bos taurus reference genome (GenBank accession number CM000177).

The genome sequencing and assembly determined that the attenuated Babesia bovis strain contains 7,962,396 bp, whereas the virulent wild-type strain contains 8,760,816 bp, which represents a difference of 9.11% in total count (Table 1). Interestingly, the GC content is slightly lower in the attenuated strain of B. bovis (41.54%), compared with the virulent strain (42.0%); in the reference genome, the GC content is 38.968% and the total length is 8.2 Mbp (13).

**TABLE 1:** Babesia bovis strains analyzed in this study and reference T2Bo

Parameter	Data for^*a*^:		
	B. bovis attenuated	B. bovis virulent	B. bovis T2Bo
No. of contigs	625	2,274	7
Total genome size (bp)	7,962,396	8,760,816	8,228,827
GC content (%)	41.54	42.0	38.96
Coverage (×)	480	130	10
*N*_50_ (bp)	172,356	100,409	1,797,577
*L*_50_	15	25	2
No. of Ns/100 kbp	0	0	NA
GenBank version no.	JAIUGG000000000.1	JAIUGF000000000.1	AAXT00000000

a All statistics are based on contigs of ≥500 bp. NA, not available.

### Data availability.

This whole-genome shotgun project has been deposited in GenBank under the accession number JAIUGF000000000 (BioSample accession number SAMN20446764 and BioProject accession number PRJNA750231) for the B. bovis virulent strain and the accession number JAIUGG000000000 (BioSample accession number SAMN20446769 and BioProject accession number PRJNA750232) for the B. bovis attenuated strain. The versions described in this paper are the first versions. The raw sequence reads have been deposited in the Sequence Read Archive (SRA) under the accession numbers SRX14134744 and SRX14130380 for the virulent and attenuated strains, respectively.
